# Role of Oxidative Stress in Periodontal Diseases

**DOI:** 10.7759/cureus.60779

**Published:** 2024-05-21

**Authors:** Ruchita T Patil, Prasad V Dhadse, Shrishti S Salian, Sanehi D Punse

**Affiliations:** 1 Department of Periodontics and Implantology, Sharad Pawar Dental College and Hospital, Datta Meghe Institute of Higher Education and Research, Wardha, IND

**Keywords:** nitric oxide (no), oral microbiota, acute inflammation, oxidative stress, periodontitis

## Abstract

Periodontal disease, a significant worldwide health burden, is characterized by chronic inflammation and destruction of periodontal tissues, including the cementum, periodontal ligament (PDL), alveolar bone, and gingival tissue. Recent research has linked the development and progression of periodontal disease to oxidative stress. This study provides comprehensive explanations of the mechanisms behind oxidative stress in periodontal disease, with a focus on the generation of reactive oxygen species (ROS) and their effects on periodontal tissues. Oxidative stress triggers a number of detrimental reactions, including lipid peroxidation, protein oxidation, and damage to deoxyribonucleic acid (DNA). Alveolar bone resorption, connective tissue degradation, and periodontal inflammation are further conditions exacerbated by these processes. In addition, the delicate balance between antioxidants and oxidants is upset by oxidative stress, which impairs antioxidant defense systems and exacerbates periodontal tissue damage. This review highlights the negative effects of oxidative stress and enhances periodontal health outcomes.

## Introduction and background

Oxygen is a crucial component for life-sustaining respiration in humans, present in the atmosphere. Ninety-five percent of oxygen is used as energy and eventually turns into water. Reactive oxygen species (ROS), also known as activated oxygen, are produced by the remaining 5% of the organism. A dysbiotic condition called periodontitis is defined by an unbalanced microbial community in the periodontal tissues, which causes persistent inflammation and the breakdown of the tooth's supporting tissues. ROS, which are mostly overproduced by hyperactive neutrophils, might damage tissues when periodontitis arises because the antioxidant defense system is unable to keep up with them. This is indicated by elevated metabolites of protein damage, deoxyribonucleic acid (DNA) damage, and lipid peroxidation. It is typically linked to polymorphonuclear leukocyte activation, which can produce ROS in an inflammatory environment [1].

Any species that can survive on its own and has one or more unpaired electrons is referred to as a free radical. The term "reactive oxygen species" (ROS) has gained popularity since it includes other reactive species that can create radicals in both intracellular and extracellular settings but are not actual radicals. Antioxidants are defined as those compounds that, in comparison to those of an oxidizable substrate, will considerably prevent or delay the substrate's oxidation when present at low concentrations [2]. Oxidative stress was defined by Sies as “a disturbance in the pro-oxidant-antioxidant balance in favor of the former, leading to potential damage” [3].

Numerous clinical and fundamental experimental research conducted in the last several years have demonstrated a robust correlation between oxidative stress and periodontitis. Gaining more knowledge about this connection will help us better understand the pathophysiology of periodontitis, which is the connection between the condition and systemic inflammation, and treatment options. Thus, the purpose of this review is to provide an overview of the most recent research on the relationship between systemic and local oxidative stress and periodontitis.

## Review

Origins of ROS and oxygen radicals

**Figure 1:**
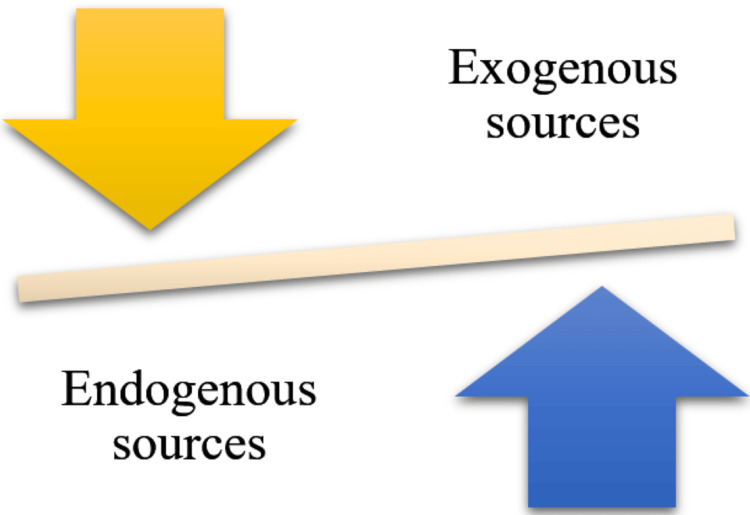
Sources of reactive oxygen species Image Credits: Ruchita T Patil

There are two types of origin of ROS (Figure 1). Exogenous sources include radiation, heat, injury, ultrasonography, UV light, exhaust fumes, ozone, smoke, infection, radiation, and exercise. The primary endogenous sources include byproducts of metabolic pathways; electron leakage from mitochondrial electron transport systems, which produces superoxide; and host defense cells (phagocytes) and connective tissue cells (fibroblasts and osteoclasts) that perform functional synthesis (maintenance, repair, and remodeling). Glycolysis is a metabolic pathway, in which glucose is broken down to produce energy. The metabolic pathway includes glycolysis. There are two glycolysis cycles: anaerobic and aerobic. During aerobic glycolysis, oxygen is consumed and utilized to form pyruvate. This cycle is carried out within the mitochondria. During this process, electrons leak from their transporters at a constant rate, reducing oxygen to the superoxide anion. It can lead to mitochondrial DNA damage. However, it is prevented by mitochondrial antioxidant scavenger’s ability to superoxide. For this reason, in vivo mitochondria represent a significant source of ROS arising from metabolism [4].

The superoxide anion (O2-) is created when oxygen is combined with one (e). When a second (e) is added, hydrogen peroxide (H2O_2_), or ROS, is formed. The hydroxyl radical (• OH) is created upon the addition of a third (e). Hydrogen (H_2_O) is created when a fourth (e) is added. 

Oxidative stresses

ROS production within cells is out of equilibrium, leading to oxidative stress, which is the body's ability to neutralize them through its antioxidant defenses. This imbalance leads to cellular damage as ROS levels rise, and the antioxidant system fails to adequately counteract their harmful effects. Intracellular ROS are typical elements of signal transduction cascades under healthy settings [5]. The balance of ROS in the body is primarily maintained by the antioxidant system. However, in inflammatory conditions, various cellular processes, such as transcription factors, protein kinases, and genomic expression, can stimulate ROS production. Proteins, lipids, and DNA are among the vital biological components that may sustain damage as a result of this elevated ROS level [3,4].

Protein Damage

The mechanisms underlying protein damage caused by ROS are intricate and not yet fully elucidated. When proteins are oxidized, they can undergo functional inactivation, which may either be reversible or irreversible. In addition, oxidized proteins become more vulnerable to degradation by proteases. During radical attacks, carbon-carbon double bonds (C=C bonds) within proteins can be affected, generating carbon-centered radical intermediates. These radicals have the potential to interact and induce structural alterations in proteins, leading to disruptions in their overall folding and functionality. The effects of ROS on proteins include folding or unfolding of proteins, which might or might not be reversible; reactions involving protein fragmentation and polymerization; breaking down of the modified protein by proteases; formation of protein radicals; formation of protein-bound ROS; and creation of stable final products, such as aldehydes or oxo-acids, which are carbonyl compounds [6].

Lipid Damage

**Figure 2:**
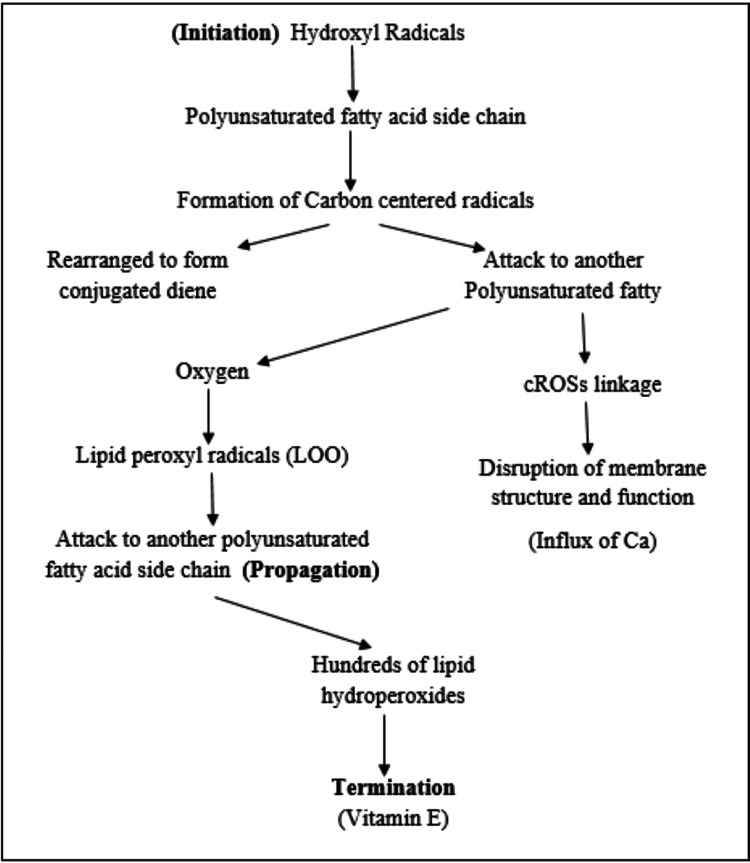
Halliwell’s reaction Image Credits: Ruchita T Patil

Lipid peroxidation, a series of events that starts when ROS attack polyunsaturated fatty acids (PUFAs) in lipid molecules, can be caused. Lipid hydroperoxides and other reactive aldehydes, including 4-hydroxynonenal (4-HNE) and malondialdehyde (MDA), are produced as a result of this process. Lipid peroxidation reaction is simplified by Halliwall in the year 1991. Halliwell’s reaction is the most important reaction of free radical species initiated by hydroxyl radical and also peroxynitrite anion. Halliwell’s reaction is described in three stages: initiation, propagation, and termination [6] (Figure 2).

During the initiation phase of lipid peroxidation, hydroxyl radicals (such as those derived from peroxynitrite) first attack the side chains of polyunsaturated fatty acids, like arachidonic acid. This leads to the formation of carbon-centered radicals, which can undergo rearrangement to form conjugated dienes or react with another polyunsaturated fatty acid. This interaction results in the cross-linking of both fatty acids, disrupting membrane structure and function, and causing an influx of calcium ions into the cell and activation of calcium-dependent proteases.

However, the most common pathway in this process involves the reaction of these radicals with oxygen, leading to the formation of lipid peroxyl radicals. These peroxyl radicals can then further attack other fatty acid side chains, continuing the chain reaction known as propagation. This chain reaction proceeds, generating numerous lipid hydroperoxides. Importantly, this destructive process can be terminated by the action of vitamin E (alpha-tocopherol), which is crucial for maintaining membrane integrity and protecting against lipid peroxidation [1].

DNA Damage

Strand breaks and base pair alterations (purine and pyrimidine bases) are two mechanisms by which peroxynitrite and hydroxyl radicals damage DNA. The nucleoside 8-hydroxydeoxyguanosine, which is tested as a marker of DNA damage, is produced when guanine is converted to 8-hydroxyguanine. Amplification of sequence, insertions, deletions, and nicking. Hydroxyl radicals cause damage to all four bases and create a characteristic “DNA fingerprint” [7]. Two different processes can lead to the formation of oxidative DNA lesions: 1) purine or pyrimidine bases directly oxidizing in DNA and 2) DNA polymerase's incorrect integration of oxidized deoxynucleoside triphosphates into DNA.

Oxidative stress and periodontitis

The role of ROS in the pathogenesis of inflammatory diseases can be described by Halliwell postulates. The four criteria proposed by Halliwell are as follows [8]: 1) The site of the injury must have ROS or oxidative damage is caused. 2) Tissue injury should happen either concurrently with or prior to the time cycle of ROS production or oxidative damage. 3) Applying ROS directly to tissues at concentrations detected in vivo during a relevant time course should replicate the damage seen in the sick tissue. 4) Because ROS have an antioxidant effect in vivo, removing or blocking their generation should reduce tissue damage to some degree.

In periodontal tissue and gingival sulcus, neutrophils are the most prevalent inflammatory cells that build up after periodontal pathogenic bacteria in biofilm induce host defense responses. It is thought that the main contributors to ROS in periodontitis are neutrophils [9]. ROS have the potential to destroy periodontal pathogens at physiological levels and act as a second messenger, mediating biological processes and conferring cytoprotective benefits. However, an oversupply of ROS can lead to a number of detrimental outcomes, such as tissue deterioration and an antagonistic cycle between the immuno-inflammatory cascade and ROS. Pathogenic bacteria and lipopolysaccharides (LPS) found in subgingival dental plaque trigger TNF-alpha and other Toll-like receptors through their DNA. Inflammatory cytokines cause the release of ROS from hyperresponsive PMNs. Activating proteins-1 and NF-kβ, on the other hand, activate osteoclasts and raise MMP (matrix metalloproteinases) concentrations, both of which lead to tissue destruction. Oxidized proteins, inflammatory mediators, and lipid peroxides are produced in excess when periodontal tissue is destroyed. These goods increase the production of ROS by further activating neutrophils, fibroblasts, and macrophages (Table 1).

**Table 1:** Effects of ROS ROS: reactive oxygen species, PG-E2: prostaglandin E2

Targeted periodontal tissue	Reaction of ROS	Effect
Ground substance	Depolymerization and degradation (non-sulfated glycosaminoglycans are more susceptible than sulfated)	All these events lead to periodontal tissue destruction and alveolar bone resorption.
Collagen	Collagenolysis	
Monocytes and macrophages	Stimulation of excessive pro-inflammatory cytokine	
Lipid peroxidation	PG-E2 production leads to bone resorption	

ROS, known for their brief existence and elusive detection, pose a challenge in assessment. However, examining ROS-related byproducts alongside enzymatic and non-enzymatic antioxidant activity offers promising avenues for evaluating the impact of oxidative stress on periodontitis pathology. Variations in oxidative stress biomarker concentrations locally correlate with periodontitis advancement, suggesting their potential utility in diagnosis and assessing therapeutic effectiveness (Table 2).

**Table 2:** Biomarkers of ROS ROS: reactive oxygen species

Biomarkers	Types of biomarkers
Biomarkers of lipid peroxidation	Conjugated dienes
	Thiobarbituric acid reactive substances (malondialdehyde)
	Isoprostanes
	Ethane/pentane and other volatile hydrocarbons
Biomarkers of DNA damage	carbohydrate moieties (deoxyribose) can be measured as biomarker
	8-hydroxydeoxyguanosine
Biomarkers of DNA damage	Protein carbonyl
	Acrolein
	Carbonyl levels

Hydrogen Peroxide (H_2_O_2_)

Hydrogen peroxide functions by generating harmful hydroxyl free radicals, which have the capacity to harm cellular structures, such as membrane lipids and DNA. The Fenton reaction is the most common reaction for generating hydroxyl radicals. It takes place between iron and hydrogen peroxide. It is highly reactive and highly toxic to living cells. Catalase, synthesized by aerobic organisms and facultative anaerobes (*Aggregatibacter actinomycetemcomitans*, *Capnocytophaga*, and *Eikenella corrodens*) and equipped with cytochrome systems, serves as a defense mechanism against metabolically generated hydrogen peroxide by converting it into harmless water and oxygen molecules [10] (Table 3).

**Table 3:** True radicals and ROS ROS: reactive oxygen species

True radicals	Reactive oxygen species (ROS)
Superoxide	Hydrogen peroxide
Hydroxyl	Hypochlorous acid
Perhydroxy	Singlet oxygen
Hydroperoxyl	Ozone
Alkoxyl	
Aryloxy	
Arylperoxyl	
Peroxyl	
Acyloxyl	
Acylperoxyl	

Hypochlorous Acid (HOCl)

HOCl's potent oxidizing capability is acknowledged for its effectiveness in eliminating pathogens. However, it also leads to considerable cellular harm, evidenced by elevated levels of lipid peroxidation, protein oxidation, and DNA damage [11].

Singlet Oxygen (1O_2_)

One of the reactive oxygen species, singlet oxygen is connected to the oxidation of low-density lipoprotein (LDL) cholesterol and the subsequent cardiovascular consequences. Polyphenol antioxidants have the ability to scavenge reactive oxygen species, lower their concentrations, and potentially stop these harmful oxidative effects. In photodynamic treatment, singlet oxygen is the active species.

Ozone (O_3_)

Ozone in physiological conditions leads to the activation of aerobic processes. It acts as an antimicrobial antioxidant agent. It acts on inflammatory response and induces synthesis of interleukins and leukotrienes. It also helps in the secretion of nitric oxide.

The role of nitric oxide synthetase

Free radicals with a brief half-life, such as nitric oxide (NO), are crucial to numerous physiological and pathological processes. Numerous mammalian cells, including those of the endothelium, neurons, smooth muscle, macrophages, neutrophils, fibroblasts, hepatocytes, chondrocytes, and synoviocytes, produce this extremely simple and reactive molecule through the oxidation of the amino acid arginine, which is known as nitric oxide synthesis (NOS). Different NOS are used to create NO.

There are three distinct isoforms of NOS: endothelial NOS (eNOS), neuronal NOS (nNOS), and inducible NOS (iNOS). The primary function of eNOS is associated with regulating vascular function, adequate NO levels are essential for maintaining normal vascular tone and blood pressure, while NO inhibits platelet adhesion and aggregation, reducing the risk of blood clots forming within blood vessels. Meanwhile, nNOS plays a crucial role in retrograde signaling across neuronal synapses. iNOS, found in various cell types like macrophages and polymorphonuclear cells, is activated in response to inflammatory stimuli, generating substantial amounts of NO over an extended period [12].

Since NO is an uncharged molecule and because of its unpaired electron, which makes it a free and stable radical, NO has biological characteristics. NO is a highly diffusible, lipophilic solute whose activities rely on its shape and concentration inside the cell. NO can affect biological mechanisms either directly or indirectly, so it has an easy penetration property. NO affects tissues in a protective, regulating, and detrimental manner. NO levels tend to be elevated in aggressive periodontitis due to increased iNOS activity in response to infection and inflammation. Elevated levels of cytokines such as interleukin-1β (IL-1β), tumor necrosis factor-alpha (TNF-α), and interleukin-6 (IL-6) are commonly observed in chronic periodontitis [13].

Antibacterial Effect

The NO produced by iNOS plays a significant role in the body's defense mechanisms against certain pathogens, as it can effectively eliminate bacteria like *Porphyromonas gingivalis* (Gram-negative, anaerobic) and *Fusobacterium nucleatum*. Nitric oxide synthetase is present in salivary glands, where NO serves as a crucial regulator of salivary vasoregulation and secretion. In the oral cavity of healthy individuals, NO production is closely linked to salivary nitrite levels, which, in turn, are influenced by dietary nitrate intake and intraoral pH levels. Research indicates that a decrease in intraoral pH is associated with an increase in NO production. Antimicrobial action against specific bacterial infections is the main beneficial impact of NO in inflammatory situations. Through the elimination of bacterial pathogens in the periodontal microenvironment, the regulated generation of NO can support the host defense system. Periodontitis-related elevations in salivary arginase activity may result in a drop in NO production below baseline. This results in a reduction of saliva's antibacterial qualities and increases the susceptibility of periodontal tissue to already-existing periodontal infections [14].

Host Tissue-Damaging Effect

The cyclooxygenase (COX-2) enzyme's inducible isoform is associated with the principal deleterious effect of NO on host tissues in periodontal disorders. During periodontitis, fibroblasts, resident, and free macrophages, but not polymorphonuclear leukocytes (PMN), exhibit a notable increase in COX-2 expression. Prostaglandins (PGs) are produced by these cell types through COX-2 activity. ROS are involved in a number of pathogenic processes, such as DNA damage, lipid peroxidation (LPO), oxidation of enzymes like antiproteases, increased apoptosis in the deepest parts of sulcular pockets, and depolymerization of extracellular matrix components [15].

In bone metabolism, NO emerges as a significant player. Bone remodeling involves a balance between osteoclast-mediated bone resorption and osteoblast-mediated bone formation. Notably, both osteoblasts and osteoclasts can generate and react to NO. The effects of NO on osteoblasts are biphasic: at high concentrations, NO inhibits osteoblast function, by reacting with superoxide anions to form peroxynitrite (ONOO−), a potent oxidant that induces nitrosative stress, damaging cellular components such as proteins, lipids, and DNA, thereby impairing osteoblast function. At low concentrations, NO can exhibit antioxidant properties, reducing oxidative stress and creating a favorable environment for osteoblast function. Despite the known effects of nitric oxide (NO) on bone in the context of inflammation, accurately predicting its impact on bone loss in periodontal diseases remains difficult [16].

## Conclusions

It is evident that the onset, progression, and severity of periodontal disease are significantly influenced by oxidative stress. The production of ROS as a result of oxidative stress triggers a range of detrimental responses, including inflammation, tissue damage, and bone resorption, all of which are indicative of periodontal disease. Moreover, oxidative stress weakens antioxidant defense mechanisms and heightens the inflammatory response locally, creating a vicious cycle that perpetuates the deterioration of periodontal tissue.

In summary, the pathophysiology of periodontal disease is largely influenced by oxidative stress, which emphasizes the significance of focused therapies including antioxidant supplements and lifestyle modifications, to lessen its harmful consequences. By treating oxidative stress, clinicians may be able to both better manage and prevent periodontal disease from developing in the first place. In the end, this will enhance the affected people's quality of life and dental health.
